# LABA/LAMA fixed-dose combinations *versus* LAMA monotherapy in the prevention of COPD exacerbations: a systematic review and meta-analysis

**DOI:** 10.1177/1753466620937194

**Published:** 2020-07-09

**Authors:** Ching-Yi Chen, Wang-Chun Chen, Chi-Hsien Huang, Yi-Ping Hsiang, Chau-Chyun Sheu, Yung-Che Chen, Meng-Chih Lin, Kuo-An Chu, Cheng-Hung Lee, Yu-Feng Wei

**Affiliations:** Division of Chest Medicine, Department of Internal Medicine, E-Da Hospital, Kaohsiung; Department of Pharmacy, E-Da Hospital, I-Shou University, Kaohsiung; Department of Family Medicine, E-Da Hospital, I-Shou University, Kaohsiung; Department of Community Healthcare & Geriatrics, Nagoya University Graduate School of Medicine, Nagoya, Aichi, Japan; Department of Pharmacy, E-Da Hospital, I-Shou University, Kaohsiung; Division of Pulmonary and Critical Care Medicine, Department of Internal Medicine, Kaohsiung Medical University Hospital, Kaohsiung; Department of Internal Medicine, College of Medicine, Kaohsiung Medical University, Kaohsiung; Division of Pulmonary and Critical Care Medicine, Kaohsiung Chang Gung Memorial Hospital and Chang Gung University College of Medicine, Kaohsiung; Division of Pulmonary and Critical Care Medicine, Kaohsiung Chang Gung Memorial Hospital and Chang Gung University College of Medicine, Kaohsiung; Division of Chest Medicine, Department of Internal Medicine, Kaohsiung Veterans General Hospital, Kaohsiung; Division of Chest Medicine, Department of Internal Medicine, National Cheng Kung University Hospital, Tainan; Department of Internal Medicine, E-Da Hospital, I-Shou University, No. 1, Yida Road, Jiao-su Village, Yan-chao District, Kaohsiung 824; School of Medicine for International Students, College of Medicine, I-Shou University, Kaohsiung

**Keywords:** chronic obstructive pulmonary disease, COPD exacerbations, LABA/LAMA FDCs, LAMA

## Abstract

**Background:**

Long-acting muscarinic antagonist (LAMA) monotherapy is recommended for chronic obstructive pulmonary disease (COPD) patients with high risk of exacerbations. It is unclear whether long-acting β2-agonist (LABA)/LAMA fixed-dose combinations (FDCs) are more effective than LAMAs alone in preventing exacerbations. The aim of this study was to systematically review the literature to investigate whether LABA/LAMA FDCs are more effective than LAMA monotherapy in preventing exacerbations.

**Methods:**

We searched several databases and manufacturers’ websites to identify relevant randomized clinical trials comparing LABA/LAMA FDC treatment with LAMAs alone ⩾24 weeks. Outcomes of interest were time to first exacerbation and rates of moderate to severe, severe and all exacerbations.

**Results:**

We included 10 trials in 9 articles from 2013 to 2018 with a total of 19,369 patients for analysis in this study. Compared with LAMA monotherapy, LABA/LAMA FDCs demonstrated similar efficacy in terms of time to first exacerbation [hazard ratio, 0.96; 95% confidence interval (CI) 0.79–1.18; *p* = 0.71], moderate to severe exacerbations [risk ratio (RR), 0.96; 95% CI 0.90–1.03; *p* = 0.28], severe exacerbations (RR, 0.92; 95% CI 0.81–1.03; *p* = 0.15), and a marginal superiority in terms of all exacerbations (RR, 0.92; 95% CI 0.86–1.00; *p* = 0.04). The incidence of all exacerbation events was lower in the LABA/LAMA FDC group for the COPD patients with a history of previous exacerbations and those with a longer treatment period (52–64 weeks).

**Conclusion:**

This study provides evidence that LABA/LAMA FDCs are marginally superior in the prevention of all exacerbations compared with LAMA monotherapy in patients with COPD.

*The reviews of this paper are available via the supplemental material section.*

## Introduction

Chronic obstructive pulmonary disease (COPD) represents a significant health burden and is currently the third leading cause of death worldwide.^1^ Long-acting bronchodilators, including long-acting β2 agonists (LABAs) and long-acting muscarinic antagonists (LAMAs), are the mainstay of treatment for symptom management in patients with COPD. In recent years, LABA/LAMA fixed-dose combinations (FDCs) have increasingly been used to treat COPD due to greater improvements in lung function, symptom scores, and health status when compared with LABA or LAMA monotherapy.^2–5^ There is solid evidence showing that LAMAs are superior to LABAs with regards to exacerbation prevention.^6^ However, the advantage of LABA/LAMA over LAMA in preventing exacerbations has not been consistently demonstrated, so the use of LABA/LAMA FDCs as initial treatment is currently guided by the level of symptoms but not the risk of exacerbations. According to the 2019 and 2020 revised Global Initiative for Chronic Obstructive Lung Disease (GOLD) reports, LAMA monotherapy is recommended as the initial choice of therapy for patients at high risk of COPD exacerbations (group C and D). LABA/LAMA FDC is an alternative choice for those with more severe symptoms. In addition, for patients with persistent exacerbations on long-acting bronchodilator monotherapy, escalation to LABA/LAMA is also recommended in current guidelines.^7^ The currently available LABA/LAMA FDCs, including olodaterol/tiotropium, indacaterol/glycopyrronium, formoterol/aclidinium, and vilanterol/umeclidinium, are widely used to treat COPD patients. However, few studies have investigated whether these LABA/LAMA FDCs offer additional benefits over LAMA monotherapy in exacerbation prevention. Recently, a large randomized controlled trial demonstrated that combining tiotropium and olodaterol did not reduce exacerbation rates as much as expected compared with tiotropium alone.^8^ Given the increased number of LABA/LAMA FDCs for clinical use in COPD patients, the initial choice of these agents or LAMA alone remains under debate in terms of exacerbation prevention. The aim of the current analysis was therefore to evaluate the comparative efficacy of LABA/LAMA FDCs and LAMA monotherapy in preventing exacerbations.

## Methods

### Search strategy and eligibility criteria

We performed a systematic literature search to identify randomized controlled trials (RCTs) evaluating the efficacy and safety of long-acting bronchodilators for COPD using PubMed, EMBASE, Cochrane Library, and Trip databases for relevant studies published up to August 1, 2019. In addition, reference lists of the included studies were scanned, and experts and physicians in this field were also consulted. This systematic review was drafted in accordance with the Preferred Reporting Items for Systematic Reviews and Meta-Analyses for Protocols (PRISMA-P) guidelines.^9^ The inclusion criteria were: (a) patients with stable COPD according to the GOLD diagnostic criteria; (b) randomized controlled trials comparing LABA/LAMA FDCs and LAMAs; (c) at least 24 weeks of treatment duration; and (d) endpoints meeting any of our outcomes of interest (time to first exacerbation, rates of moderate to severe, severe and all exacerbations). We also included studies with subgroup analysis comparing LAMA/LABA FDCs with individual LAMA components.

### Data extraction and risk of bias assessment

The title and abstract of each study were independently assessed by three authors (C-YC, W-CC, and Y-FW) to confirm the eligibility for analysis, and any difference in opinion was resolved by consensus. Data from the included studies were independently extracted and checked by C-YC and Y-FW Two reviewers (C-YC and W-CC) independently assessed the risk of bias of the included studies according to the recommendations in the Cochrane Handbook for Systematic Reviews of Interventions 5.1. Disagreements were resolved by consensus or assessed by other authors (Y-PH and C-HH).

### Outcomes of interest

The outcomes of interest were the frequency of acute exacerbations (time to first exacerbation, rates of moderate to severe, severe and all exacerbations). Frequencies of exacerbations were also analyzed according to the treatment duration, high-risk *versus* low-risk populations, and tiotropium *versus* non-tiotropium groups.

### Statistical analysis

Studies were pooled using risk ratios (RRs) for dichotomous outcomes and hazard ratios (HRs) for time to event outcomes in random effect models, respectively.^10^ A 95% confidence interval (CI) was set to determine statistical significance. Between-study heterogeneity assessed using the *I*^2^ test was considered to be moderate to high at a *p*-value < 0.10 and *I*^2^ value > 50%. Publication bias was examined using eyeballing, funnel plots and Egger’s test. Sensitivity analyses were performed to assess the contribution of each study to the pooled estimate by excluding individual studies one at a time and recalculating the pooled HR estimates for the remaining studies (one-study-removed meta-analysis). For any three-arm trials (e.g. indacaterol/glycopyrronium *versus* glycopyrronium *versus* tiotropium), each pairwise comparison (i.e. indacaterol/glycopyrronium *versus* glycopyrronium, and indacaterol/glycopyrronium *versus* tiotropium) was used in the meta-analysis by dividing the sample size in half to match the total sample size when adding together. High-risk *versus* low-risk exacerbation patient groups were defined according to the previous exacerbation history (⩾1 *versus* no exacerbations) or lung function (⩾50% *versus* <50% of forced expiratory volume in the first second) for the majority patients in the study. The meta-analysis was performed using Review Manager Software version 5.3 (Cochrane Library Software, Oxford, UK).

## Results

### Study characteristics

**Figure 1.**
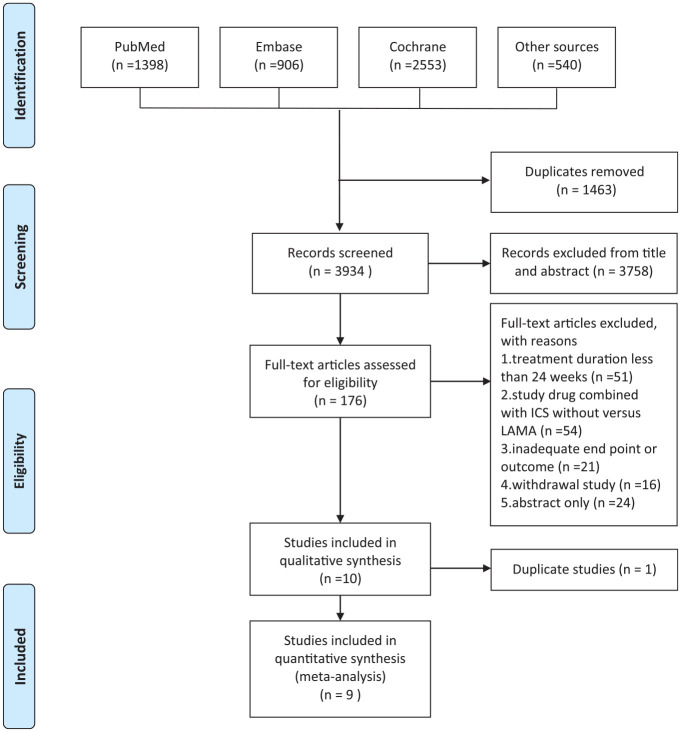
Flow diagram of study identification. ICS, inhaled corticosteroids; LAMA, long-acting muscarinic antagonist.

The relevant research and studies are shown in Figure 1. Two articles reported different outcomes from the same patients group so only one was included for analysis.^11,12^ Finally, a total of 19,369 patients were included in nine published articles from 2013 to 2018.^8,12–19^ Among these included articles, two compared indacaterol/glycopyrronium, glycopyrronium, and tiotropium; two compared tiotropium/olodaterol and tiotropium; two compared aclidinium/formoterol and aclidinium; one compared umeclidinium/vilanterol, umeclidinium, and tiotropium; one compared umeclidinium/vilanterol and tiotropium; and one compared glycopyrrolate/formoterol fumarate, glycopyrrolate, and tiotropium.

All the studies were RCTs and double-blinded, with a treatment period from 24 to 64 weeks. Three of the articles reported outcomes from two duplicate RCTs, and one of them reported outcomes of two trials separately.^12,15,18^ We paired the LAMA/LABA FDCs and compared them with individual or different LAMA components in the studies including three or more comparators. The characteristics of the included studies are summarized in Table 1.

**Table 1.** Study characteristics of the included studies.

Study	Study duration (weeks)	Number of patients	Treatment comparison/dose (µg)	Mean age (years)	Male (%)	Baseline FEV_1_ % predicted	COPD severity by lung function	Exacerbation risk by lung function	Exacerbation history in previous year	Exacerbation risk by previous AE
Bateman *et al.*^13^	26	474	Indacaterol 110/glycopyrronium 50	64.0	76.4	55.7	Moderate (63.8%) Severe (36.1%)	Low	0 (73.7%) 1 (19.5%) ⩾2 (6.8%)	Low
		473	Glycopyrronium 50	64.3	77.2	55.1				
		480	Tiotropium 18	63.5	75.0	55.1				
Wedzicha *et al.*^14^	64	729	Indacaterol 110/glycopyrronium 50	63.1	76.0	37.0	Severe (79.2%) Very severe (20.8%)	High	0 (1.5%) 1 (76.5%) ⩾2 (21.4%)	High
		740	Glycopyrronium 50	63.1	73.0	37.3				
		737	Tiotropium 18	63.6	75.0	37.4				
Decramer *et al.*^15^ study 1	24	212	Umeclidinium 62.5/vilanterol 25	63.0	70.0	48.0	Moderate (48.0%) Severe (41.2%) Very severe (10.8%)	High	0 (51.2%) ⩾1 (48.8%)	Low
		208	Tiotropium 18	62.6	67.0	47.8				
Decramer *et al.*^15^ study 2	24	217	Umeclidinium 62.5/vilanterol 25	65.0	65.0	47.7	Moderate (43.9%) Severe (43.2%) Very severe (12.8%)	High	0 (66.5%) ⩾1 (33.5%)	Low
		222	Umeclidinium 125	64.5	67.0	46.2				
		215	Tiotropium 18	65.2	71.0	47.4				
Maleki-Yazdi *et al*.^16^	24	454	Umeclidinium 62.5/vilanterol 25	61.9	68.0	46.2	Moderate (41.4%) Severe (45.6%) Very severe (12.9%)	High	N/A	N/A
		451	Tiotropium 18	62.7	67.0	46.5				
Singh *et al.*^17^	24	385	Aclidinium 400/formoterol 12	62.7	67.8	54.6	Moderate (59.2%) Severe (40.8%)	Low	N/A	N/A
		385	Aclidinium 400	63.1	66.5	53.6				
Buhl *et al*.^12^	52	1029	Tiotropium 5/olodaterol 5	63.8	71.2	49.3	Moderate (49.4%) Severe (38.6%) Very severe (12.0%)	High	N/A	N/A
		1033	Tiotropium 5	63.9	73.1	49.7				
Hanania *et al.*^18^	52	1035	Glycopyrrolate 18/formoterol 9.6	62.7	54.3	43.4	Very severe (10.2%)	N/A	0 (83.3%) 1 (9.5%) ⩾2 (7.2%)	Low
		888	Glycopyrrolate 18	62.8	55.9	42.6				
		450	Tiotropium 18	62.9	59.6	42.7				
D’Urzo *et al.*^19^	52	335	Aclidinium 400/formoterol 12	64.2	50.1	53.2	Moderate (56.3%) Severe (43.7%)	Low	0 (79.2%) ⩾1 (20.8%)	Low
		337	Aclidinium 400	64.4	55.8	53.0				
Calverley *et al*.^8^	52	3939	Tiotropium 5/olodaterol 5	66.5	71.0	44.6	N/A	N/A	1 (55.7%) ⩾2 (44.3%)	High
		3941	Tiotropium 5	66.3	72.0	44.5				

AE, acute exacerbations; COPD, chronic obstructive pulmonary disease; FEV_1_, forced expiratory volume in the first second.

### Risk of bias

**Figure 2.**
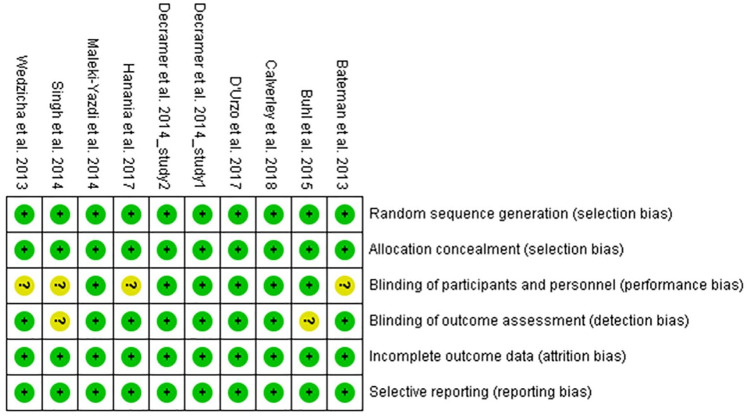
Risk of bias graph: review authors’ judgement about the risk of each item of bias presented as percentages across all included studies.

Risk of bias assessments for the included studies were performed by the review authors independently. Most of the studies had a low risk of bias as shown by sufficient evidence of random sequence generation, double blinding protocol, and complete outcome assessment (Figure 2). However, an unclear risk of performance bias was found in four studies due to not mentioning the double dummy technique,^13,14,17,18^ and detection bias may have occurred in two studies due to incomplete descriptions of the blinding of outcome assessments.^12,17^

### Outcome assessments

#### Time to first exacerbation

**Figure 3.**
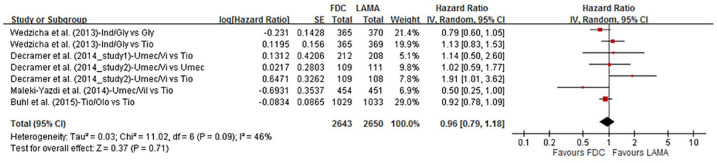
Forrest plot comparing LABA/LAMA FDCs and LAMAs on time to first exacerbation. CI, confidence interval; FDC, fixed-dose combination; LABA, long-acting β2-agonist; LAMA, long-acting muscarinic antagonist.

Four publications (including five RCTs, *n* = 5293) reported the time to first exacerbation as the endpoint (Figure 3). There was no statistical difference between the patients receiving LAMA/LABA FDCs compared with individual LAMAs (tiotropium, umeclidinium, and glycopyrronium). The HR for an exacerbation was 0.96 (95% CI 0.79–1.18; *p* = 0.71, *I*^2^ = 46%). Subgroup analyses according to different LAMAs (tiotropium and non-tiotropium), treatment duration (24 weeks and 52–64 weeks), and risk of exacerbations (by exacerbation history) were all not statistically significant (Supplemental Figures S1, S2, and S3).

#### Moderate-to-severe exacerbations

**Figure 4.**
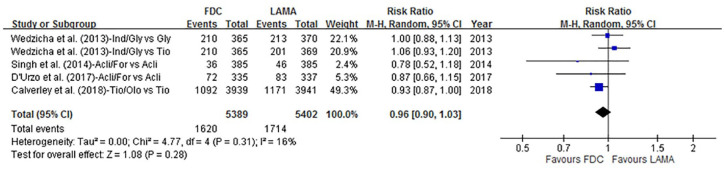
Forrest plot comparing LABA/LAMA FDCs and LAMAs with events of moderate-to-severe exacerbations. CI, confidence interval; FDC, fixed-dose combination; LABA, long-acting β2-agonist; LAMA, long-acting muscarinic antagonist.

Moderate-to-severe exacerbation data were available in four articles (*n* = 10,791). Overall, 30.0% (1620/5389) of the patients receiving LABA/LAMA FDCs experienced moderate-to-severe exacerbations, compared with 31.7% (1714/5402) of the patients receiving LAMAs alone (Figure 4). The RR was 0.96 (95% CI 0.90–1.03; *p* = 0.28, *I*^2^ = 16%), and no statistical difference was found. We then analyzed LABA/LAMA FDCs compared with different LAMAs (tiotropium and non-tiotropium), treatment duration (24 weeks and 52–64 weeks), and risk of exacerbations (by exacerbation history), but no statistically significant differences were found (Supplemental Figures S4, S5, and S6).

#### Severe exacerbations

**Figure 5.**
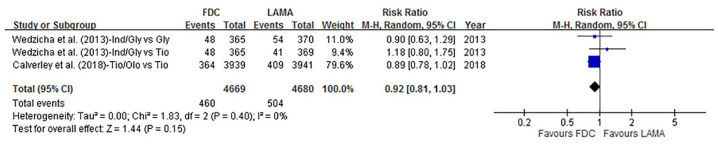
Forrest plot comparing LABA/LAMA FDCs and LAMAs with events of severe exacerbations. CI, confidence interval; FDC, fixed-dose combination; LABA, long-acting β2-agonist; LAMA, long-acting muscarinic antagonist.

Only two publications (*n* = 9349) reported severe exacerbations as one of the endpoints. There was no statistical difference between the LABA/LAMA FDCs and LAMA groups in terms of severe exacerbations [9.9% (460/4669) *versus* 10.8% (504/4680), respectively], with an RR of 0.92 (95% CI 0.81–1.03, *p* = 0.15, *I*^2^ = 0%) (Figure 5).

#### All exacerbations

**Figure 6.**
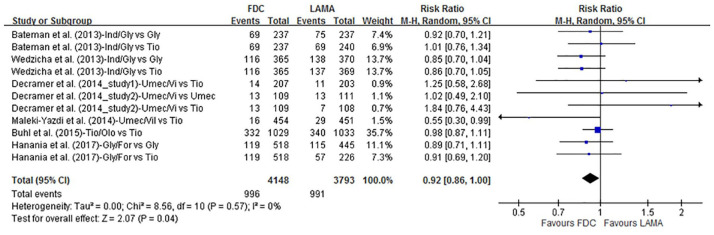
Forrest plot comparing LABA/LAMA FDCs and LAMAs with events of all exacerbations. CI, confidence interval; FDC, fixed-dose combination; LABA, long-acting β2-agonist; LAMA, long-acting muscarinic antagonist.

The incidence of all exacerbations from six articles (including 9 RCTs, *n* = 7941) was lower in those treated with LABA/LAMA FDCs than in those treated with LAMAs [24.0% (996/4148) *versus* 26.1% (991/3799), respectively], with an RR of 0.92 (95% CI 0.86–1.00; *p* = 0.04, *I*^2^ = 0%) (Figure 6). Subgroup analyses showed similar efficacy in those treated with LABA/LAMA FDCs compared with those treated with different LAMAs, but slight superiority was demonstrated in those with a longer treatment duration (52–64 weeks) (RR, 0.92; 95% CI 0.85–1.00; *p *= 0.04) (Supplemental Figures S7 and S8). Other analyses according to the risk of exacerbations (high-risk *versus* low-risk, stratified by exacerbation history or lung function), demonstrated a lower rate of all exacerbations only in the high-risk population stratified by exacerbation history (RR, 0.85; 95% CI 0.74–0.98; *p *= 0.03) (Supplemental Figures S9 and S10).

## Discussion

**The results of this meta-analysis demonstrated that** LABA/LAMA FDCs were marginally beneficial in the prevention of all exacerbations compared with LAMA monotherapy in COPD patients. According to the revised GOLD reports, LAMA monotherapy is preferred as the initial treatment choice in group C and D COPD patients, and LABA/LAMA FDCs are an alternative choice for those with more symptoms.^7^ Escalation to LABA/LAMA is also recommended for patients with persistent exacerbations on LAMA monotherapy. Nevertheless, the 2019 updated National Institute for Health and Clinical Excellence (NICE) guidelines recommended LAMA+LABA dual therapy, but not monotherapy, as the preferred initial treatment in stable COPD patients who remain breathless or have exacerbations despite optimal non-pharmacological management and after using a short-acting bronchodilator.^20^ The NICE guidelines also state that LAMA/LABA dual therapy provides the greatest benefit to overall quality of life compared with monotherapy, and that dual therapy is better than other inhaled treatments for many individual outcomes (such as reducing the risk of moderate-to-severe exacerbations) and is the most cost-effective option. Nevertheless, the currently available evidence is not consistent with regards to whether LABA/LAMA combination therapy is more effective than LAMA monotherapy in preventing exacerbations.

Previous meta-analyses have focused on comparisons of LABA/LAMA combinations and LAMA monotherapy. One meta-analysis conducted by Rodrigo *et al.* demonstrated greater efficacy and comparable safety profiles with LABA/LAMA combinations *versus* LAMAs. However, the COPD exacerbation rate was not reported due to insufficient data.^21^ Rogliani *et al*. assessed the evidence for LABA/LAMA FDCs in the treatment of COPD. They analyzed trial results of the available LABA/LAMA FDCs and found that only indacaterol/glycopyrronium demonstrated superiority to glycopyrronium, but that there was no statistically significant difference *versus* tiotropium.^22^

Oba *et al.* conducted a comprehensive Cochrane review of different groups of inhalers (including LABA/LAMA combinations and LAMA monotherapy) in patients with moderate-to-severe COPD. They analyzed eight studies comparing different LABA/LAMA combinations and LAMA monotherapy, and demonstrated no statistical differences in terms of severe exacerbations [odds ratio (OR) 0.90; 95% CI 0.59–1.36; *p* = 0.61] and moderate-to-severe exacerbations (OR 0.96, CI 0.75–1.23; *p* = 0.77).^23^ Although this finding is consistent with our results, Oba’s review enrolled two studies with LABA/LAMA combinations in two separate inhalers and one study which was a 12-week study that was inappropriate to evaluate exacerbation outcomes. In addition, the largest DYNAGITO trial was not included for analysis in their review.

Farne *et al*. reviewed 10 trials comparing tiotropium plus a LABA to tiotropium or a LABA alone and concluded that adding tiotropium to a LABA reduced exacerbations, but that adding a LABA to tiotropium did not.^24^ Another comprehensive systematic review reported by Halpin *et al*. demonstrated that tiotropium was beneficial in reducing the risk of exacerbations compared with other maintenance treatments. Their analysis showed that tiotropium provided similar efficacy to glycopyrronium and a LABA/LAMA FDC (glycopyrronium/indacaterol), although not all studies were sufficiently powered to demonstrate differences in exacerbation outcomes.^25^ However, the large DYNAGITO trial reported that a combination of tiotropium and olodaterol failed to reduce the exacerbation rate as expected when compared with tiotropium alone.^8^ Given the conflicting results, we stratified and analyzed different LAMAs as tiotropium and non-tiotropium. Our analysis showed that exacerbation events in those receiving LAMA/LABA FDCs were not significantly different compared with those receiving tiotropium or non-tiotropium therapy.

In the current study, we also compared LABA/LAMA FDC and LAMA therapy in patients grouped by the risk of exacerbations and treatment duration. We analyzed the COPD patients with a high or low risk of exacerbations in the studies according to lung function or previous exacerbation history. Our results demonstrated that only the incidence of all exacerbation (but not severe or moderate to severe exacerbation) events was lower in the LABA/LAMA FDCs for COPD patients with a history of previous exacerbation. However, this result was from the SPARK study in which most of the patients had a previous history of exacerbation (DYNAGITO study did not provide outcomes of all exacerbation).^14^ In addition, LABA/LAMA FDCs showed a small benefit in the incidence of all exacerbation events for those with a longer treatment period (52–64 weeks compared with 24–26 weeks), probably due to the long-term effects of LABA/LAMA combination therapy in terms of lung function improvements.

The GOLD reports recommend escalating to LABA/LAMA treatment for patients with persistent dyspnea or exacerbations on long-acting bronchodilator monotherapy. This escalation strategy is supported in part by this systematic review, in that LABA/LAMA FDCs may provide better efficacy in the prevention of all exacerbations compared to LAMA monotherapy, although the incremental benefit was small.

There are several limitations to this study. First, the results of this meta-analysis could not indicate differences between the various LABA/LAMA FDCs and LAMAs (although we stratified the LAMA group by tiotropium and non-tiotropium), and their efficacy may not be exactly the same. Second, assessments of the high- and low-risk patients in the included studies were not consistent, even though we stratified the majority of the study patients by lung function or previous exacerbation history. Third, exacerbation type (requiring antibiotic or systemic steroid therapy) was not analyzed due to insufficient data in the included studies.

In conclusion, our meta-analysis suggests that LAMA/LABA FDCs produce a small benefit in the prevention of all exacerbations compared to LAMA monotherapy, but similar efficacy in terms of time to first exacerbation, the rate of moderate-to-severe, and severe exacerbations. In addition to greater improvements in lung function, symptom scores, and health status, our findings provide evidence that LABA/LAMA FDCs are also better than LAMA monotherapy in terms of all exacerbation prevention and could be considered as the first-line treatment for COPD patients, especially in those with a history of previous exacerbations.

## Supplemental Material

Author_Response_1 – Supplemental material for LABA/LAMA fixed-dose combinations versus LAMA monotherapy in the prevention of COPD exacerbations: a systematic review and meta-analysis Click here for additional data file. Supplemental material, Author_Response_1 for LABA/LAMA fixed-dose combinations versus LAMA monotherapy in the prevention of COPD exacerbations: a systematic review and meta-analysis by Ching-Yi Chen, Wang-Chun Chen, Chi-Hsien Huang, Yi-Ping Hsiang, Chau-Chyun Sheu, Yung-Che Chen, Meng-Chih Lin, Kuo-An Chu, Cheng-Hung Lee and Yu-Feng Wei in Therapeutic Advances in Respiratory Disease

Author_Response_2 – Supplemental material for LABA/LAMA fixed-dose combinations versus LAMA monotherapy in the prevention of COPD exacerbations: a systematic review and meta-analysis Click here for additional data file. Supplemental material, Author_Response_2 for LABA/LAMA fixed-dose combinations versus LAMA monotherapy in the prevention of COPD exacerbations: a systematic review and meta-analysis by Ching-Yi Chen, Wang-Chun Chen, Chi-Hsien Huang, Yi-Ping Hsiang, Chau-Chyun Sheu, Yung-Che Chen, Meng-Chih Lin, Kuo-An Chu, Cheng-Hung Lee and Yu-Feng Wei in Therapeutic Advances in Respiratory Disease

Reviewer_1_v.1 – Supplemental material for LABA/LAMA fixed-dose combinations versus LAMA monotherapy in the prevention of COPD exacerbations: a systematic review and meta-analysis Click here for additional data file. Supplemental material, Reviewer_1_v.1 for LABA/LAMA fixed-dose combinations versus LAMA monotherapy in the prevention of COPD exacerbations: a systematic review and meta-analysis by Ching-Yi Chen, Wang-Chun Chen, Chi-Hsien Huang, Yi-Ping Hsiang, Chau-Chyun Sheu, Yung-Che Chen, Meng-Chih Lin, Kuo-An Chu, Cheng-Hung Lee and Yu-Feng Wei in Therapeutic Advances in Respiratory Disease

Reviewer_1_v.2 – Supplemental material for LABA/LAMA fixed-dose combinations versus LAMA monotherapy in the prevention of COPD exacerbations: a systematic review and meta-analysis Click here for additional data file. Supplemental material, Reviewer_1_v.2 for LABA/LAMA fixed-dose combinations versus LAMA monotherapy in the prevention of COPD exacerbations: a systematic review and meta-analysis by Ching-Yi Chen, Wang-Chun Chen, Chi-Hsien Huang, Yi-Ping Hsiang, Chau-Chyun Sheu, Yung-Che Chen, Meng-Chih Lin, Kuo-An Chu, Cheng-Hung Lee and Yu-Feng Wei in Therapeutic Advances in Respiratory Disease

Reviewer_2_v.1 – Supplemental material for LABA/LAMA fixed-dose combinations versus LAMA monotherapy in the prevention of COPD exacerbations: a systematic review and meta-analysis Click here for additional data file. Supplemental material, Reviewer_2_v.1 for LABA/LAMA fixed-dose combinations versus LAMA monotherapy in the prevention of COPD exacerbations: a systematic review and meta-analysis by Ching-Yi Chen, Wang-Chun Chen, Chi-Hsien Huang, Yi-Ping Hsiang, Chau-Chyun Sheu, Yung-Che Chen, Meng-Chih Lin, Kuo-An Chu, Cheng-Hung Lee and Yu-Feng Wei in Therapeutic Advances in Respiratory Disease

Supplemental_Figures – Supplemental material for LABA/LAMA fixed-dose combinations versus LAMA monotherapy in the prevention of COPD exacerbations: a systematic review and meta-analysis Click here for additional data file. Supplemental material, Supplemental_Figures for LABA/LAMA fixed-dose combinations versus LAMA monotherapy in the prevention of COPD exacerbations: a systematic review and meta-analysis by Ching-Yi Chen, Wang-Chun Chen, Chi-Hsien Huang, Yi-Ping Hsiang, Chau-Chyun Sheu, Yung-Che Chen, Meng-Chih Lin, Kuo-An Chu, Cheng-Hung Lee and Yu-Feng Wei in Therapeutic Advances in Respiratory Disease
