# Supplementary material for: LABA/LAMA fixed-dose combinations versus LAMA monotherapy in the prevention of COPD exacerbations: a systematic review and meta-analysis
Source: Ther Adv Respir Dis. 2020 Jul 9;14:1753466620937194. doi: 10.1177/1753466620937194 (PMC7350046; doi:10.1177/1753466620937194)
Supplement: Reviewer_1_v.1 – Supplemental material for LABA/LAMA fixed-dose combinations versus LAMA monotherapy in the prevention of COPD exacerbations: a systematic review and meta-analysis [file Reviewer_1_v.1.pdf]

Reviewer 1 v. 1

Comments to the Author

Methods:

- Authors should describe their detailed search strategies. It is very surprising that they identified more manuscripts from the Cochrane Library, less from Pubmed and even less from Embase.

- Was the SR protocol submitted to the PROSPERO register prospectively? It appears the study was started in April 2019 and systematic searches were conducted in August 2019 (3 months prior to registration).

- "For any three-arm trials (e.g., indacaterol/glycopyrronium versus glycopyrronium versus tiotropium), each pairwise comparison (i.e., indacaterol/glycopyrronium versus glycopyrronium, and indacaterol/glycopyrronium versus tiotropium) was used in the meta-analysis by dividing the sample size in half". The sample of the control group should be devided in half, not the overall sample size. Please revise

- "All systematic review protocols were registered on PROSPERO with a publicly available database". Please rephrase.

Results:

- In figure 1, authors state they identified 1,463 records after duplicates removed, but then state that 3,934 were screened. Could they explain?

- Subgroup analyses of studies evaluating high-/low- exacerbation risk populations are the most pertinent and are not presented in forest plots. Also, authors only conducted this subgroup analysis for the outcome "all exacerbations". They should evaluate it for all outcomes.

- Actually, subgroup analyses are not clearly presented in the manuscript. Each for the subgroup analyses should be presented in the relevant section describing each outcome in the results.

Discussion:

- Authors stated that only SPARK trial recruited patients with a history of exacerbations. However, that was also true for the DYNAGITO study. This will need to be taken in to consideration in the subgroup analyses.

- The discussion needs to be revised. There is a bit of repetition and poor organization. Also, when judging the GOLD and NICE recommendations, authors should also consider the impact of LABA/LAMA vs LAMA monotherapy on symptoms and health status. Data on these may be found in the following meta-analyses: Miravitlles et al. Efficacy and safety of tiotropium and olodaterol in COPD: a systematic review and meta-analysis. Respir Res 2017. AND Ni et al. Combined aclidinium bromide and long-acting beta2-agonist for chronic obstructive pulmonary disease (COPD). Cochrane Database Syst Rev 2018.
