# Supplementary material for: LABA/LAMA fixed-dose combinations versus LAMA monotherapy in the prevention of COPD exacerbations: a systematic review and meta-analysis
Source: Ther Adv Respir Dis. 2020 Jul 9;14:1753466620937194. doi: 10.1177/1753466620937194 (PMC7350046; doi:10.1177/1753466620937194)
Supplement: Reviewer_1_v.2 – Supplemental material for LABA/LAMA fixed-dose combinations versus LAMA monotherapy in the prevention of COPD exacerbations: a systematic review and meta-analysis [file Reviewer_1_v.2.pdf]

Reviewer 1 v.2

Comments to the Author

Authors have now addressed most of the peer review comments. There are small grammatical and syntax errors that will be likely picked up during copy-editing.

My only suggestion to the authors is to document at which study of the review was the PROSPERO protocol submitted (e.g. this systematic review protocol was registered with PROSPERO before commencing data extraction // or before conducting the meta-analyses). If the protocol was submitted after the meta-analyses were conducted, it is not a prospective protocol and authors should remove it.
