# Supplementary material for: LABA/LAMA fixed-dose combinations versus LAMA monotherapy in the prevention of COPD exacerbations: a systematic review and meta-analysis
Source: Ther Adv Respir Dis. 2020 Jul 9;14:1753466620937194. doi: 10.1177/1753466620937194 (PMC7350046; doi:10.1177/1753466620937194)
Supplement: Reviewer_2_v.1 – Supplemental material for LABA/LAMA fixed-dose combinations versus LAMA monotherapy in the prevention of COPD exacerbations: a systematic review and meta-analysis [file Reviewer_2_v.1.pdf]

Reviewer 2 v.1

Comments to the Author

The authors present the results of a systematic review and meta-analysis of efficacy of LABA/LAMA versus LAMA in prevention of exacerbations of COPD. The topic is of interest and the study is overall well conducted. However, there are a series of issues that should be addressed by the authors.

1. Abstract. The conclusion should be downgraded. It is now too strong compared to the poor results reported.

Results (page 11, line 43). It reads that two studies reported different results for the same population (Refs. 10 and 11) and only one was selected for inclusion. Can the authors expand on this? What was the difference? Which one was selected and why?

Results. In general, more emphasis is required for analysis in patients at high risk for exacerbations. This subanalysis should include patients with previous exacerbations (irrespective of lung function impairment).

Discussion (Page 15, line 19). GOLD is not a guideline, please rephrase.

Discussion (Page 18, line 16). The text reads that the only study including patients at high risk of exacerbations was SPARK, but DYNAGITO also included patients with at least one exacerbation the previous year and almost half of them with 2 or more or 1 severe.

Table 1. For the Hanania's study the frequency of patients with 0 exacerbations is missing.

Table 1. The description of DYNAGITO (Calverley et al) is wrong. It reads that 55.5% of patients had 0 exacerbations the previous year, but, by protocol, all included patents had to have at least 1 exacerbation the previous year. The 55.5% correspond to patients who had only one moderate exacerbation and the remaining 44.5% had either 2 or more moderate or at least 1 severe exacerbation. Therefore, when performing the subanalysis of patients with high risk of exacerbations the authors must include the DYNAGITO participants and re-run the analysis.
