# Supplementary figures and images for: LABA/LAMA fixed-dose combinations versus LAMA monotherapy in the prevention of COPD exacerbations: a systematic review and meta-analysis
Source: Ther Adv Respir Dis. 2020 Jul 9;14:1753466620937194. doi: 10.1177/1753466620937194 (PMC7350046; doi:10.1177/1753466620937194)

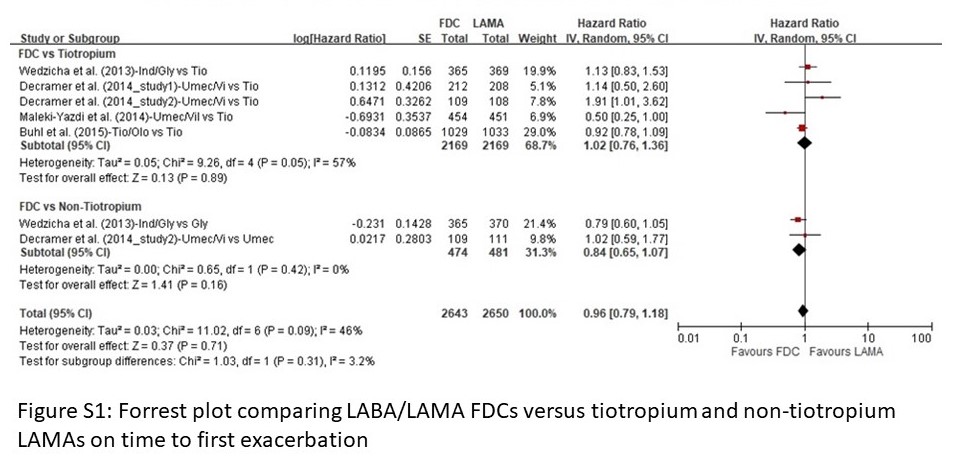

Supplement: Supplemental_Figures – Supplemental material for LABA/LAMA fixed-dose combinations versus LAMA monotherapy in the prevention of COPD exacerbations: a systematic review and meta-analysis [file Supplemental_Figures.zip › Figure S1.jpg]

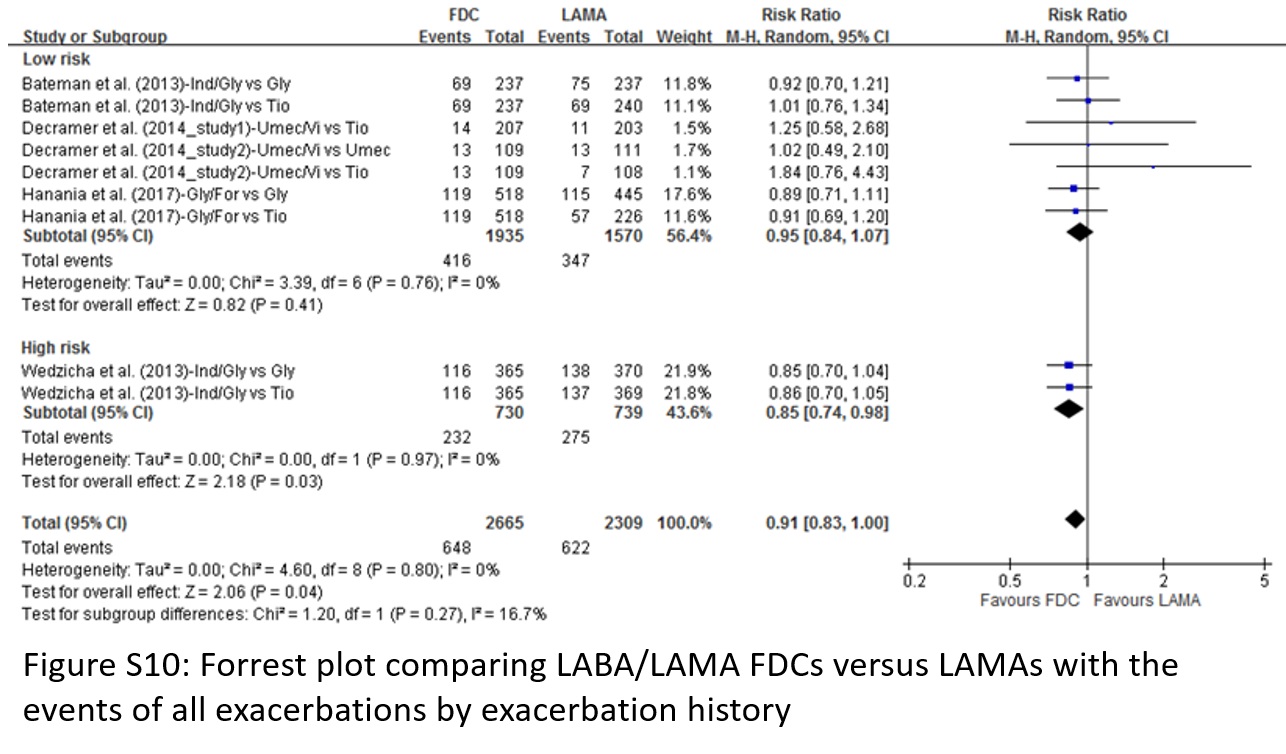

Supplement: Supplemental_Figures – Supplemental material for LABA/LAMA fixed-dose combinations versus LAMA monotherapy in the prevention of COPD exacerbations: a systematic review and meta-analysis [file Supplemental_Figures.zip › Figure S10.jpg]

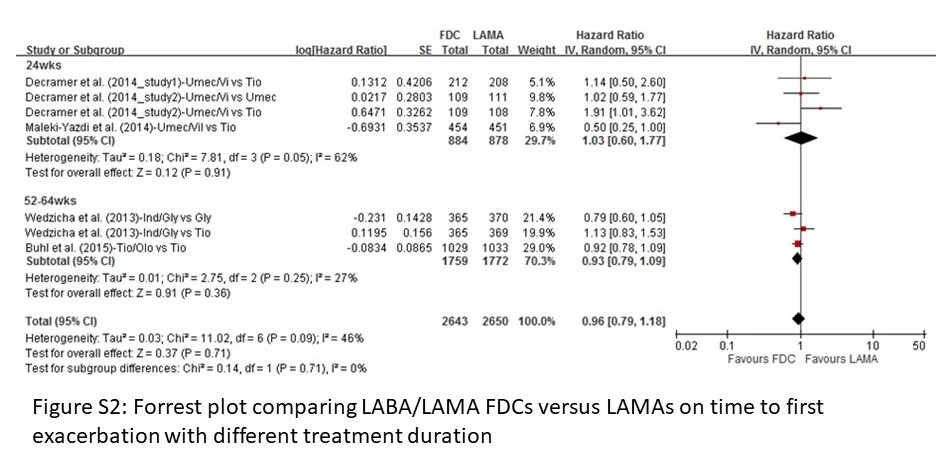

Supplement: Supplemental_Figures – Supplemental material for LABA/LAMA fixed-dose combinations versus LAMA monotherapy in the prevention of COPD exacerbations: a systematic review and meta-analysis [file Supplemental_Figures.zip › FIgure S2.jpg]

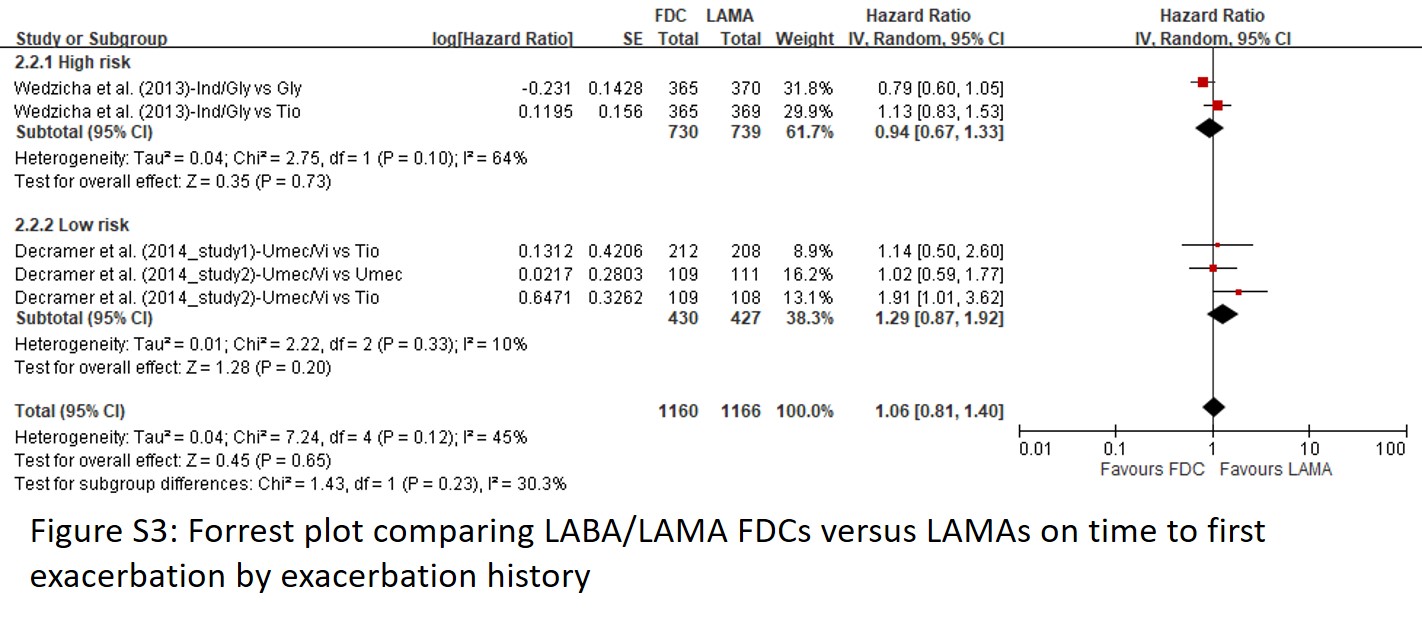

Supplement: Supplemental_Figures – Supplemental material for LABA/LAMA fixed-dose combinations versus LAMA monotherapy in the prevention of COPD exacerbations: a systematic review and meta-analysis [file Supplemental_Figures.zip › Figure S3.jpg]

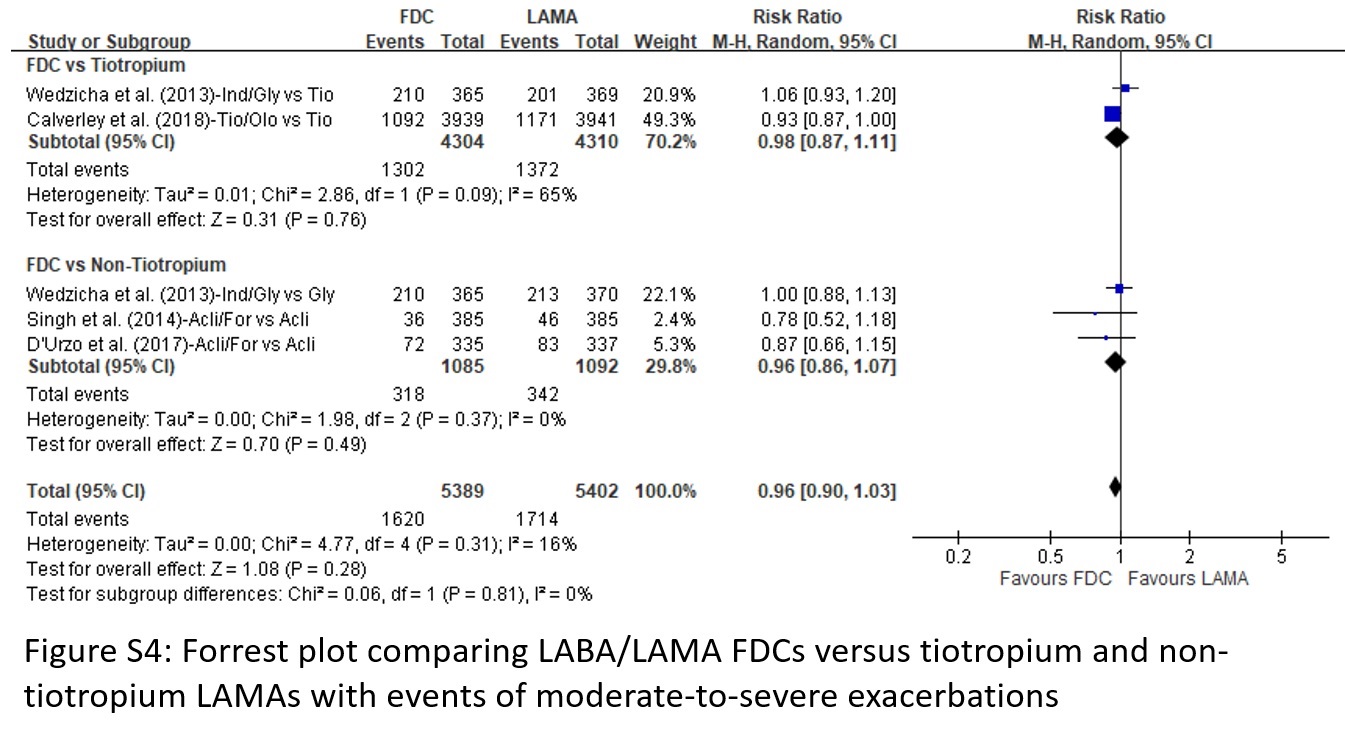

Supplement: Supplemental_Figures – Supplemental material for LABA/LAMA fixed-dose combinations versus LAMA monotherapy in the prevention of COPD exacerbations: a systematic review and meta-analysis [file Supplemental_Figures.zip › Figure S4.jpg]

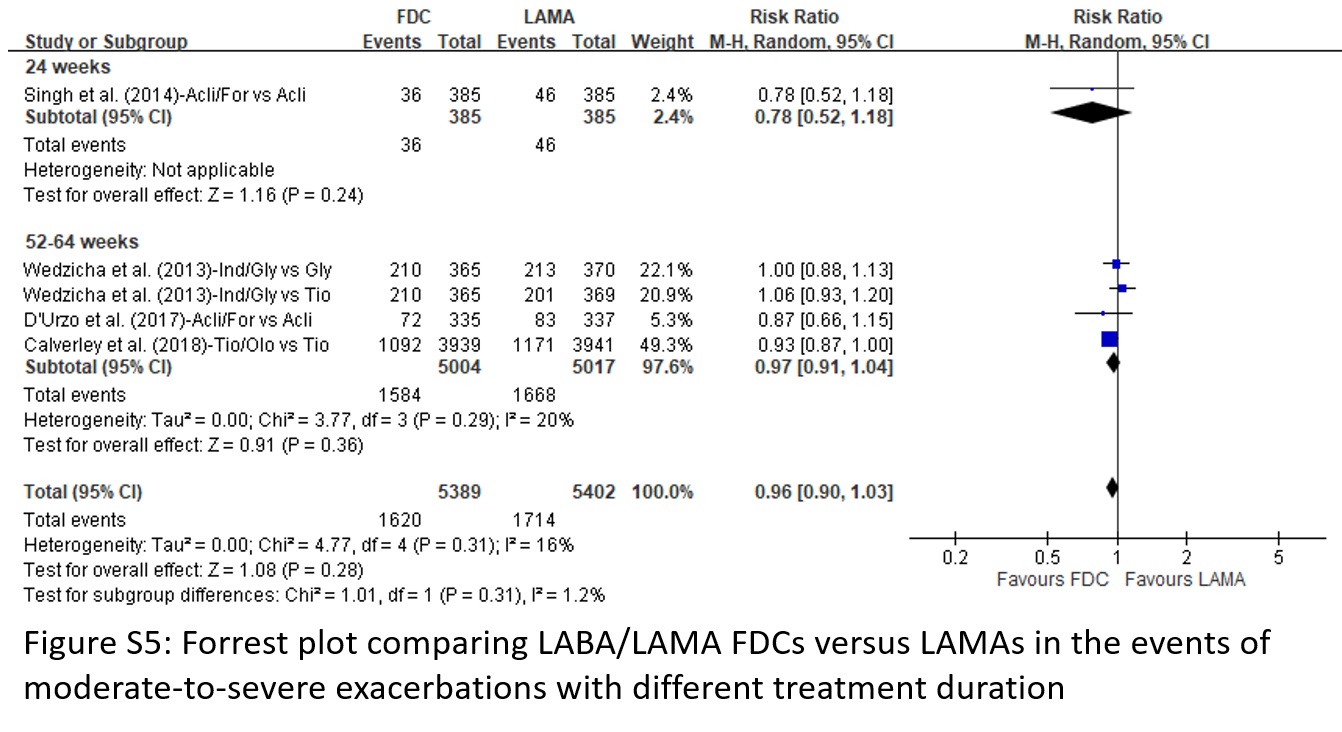

Supplement: Supplemental_Figures – Supplemental material for LABA/LAMA fixed-dose combinations versus LAMA monotherapy in the prevention of COPD exacerbations: a systematic review and meta-analysis [file Supplemental_Figures.zip › Figure S5.jpg]

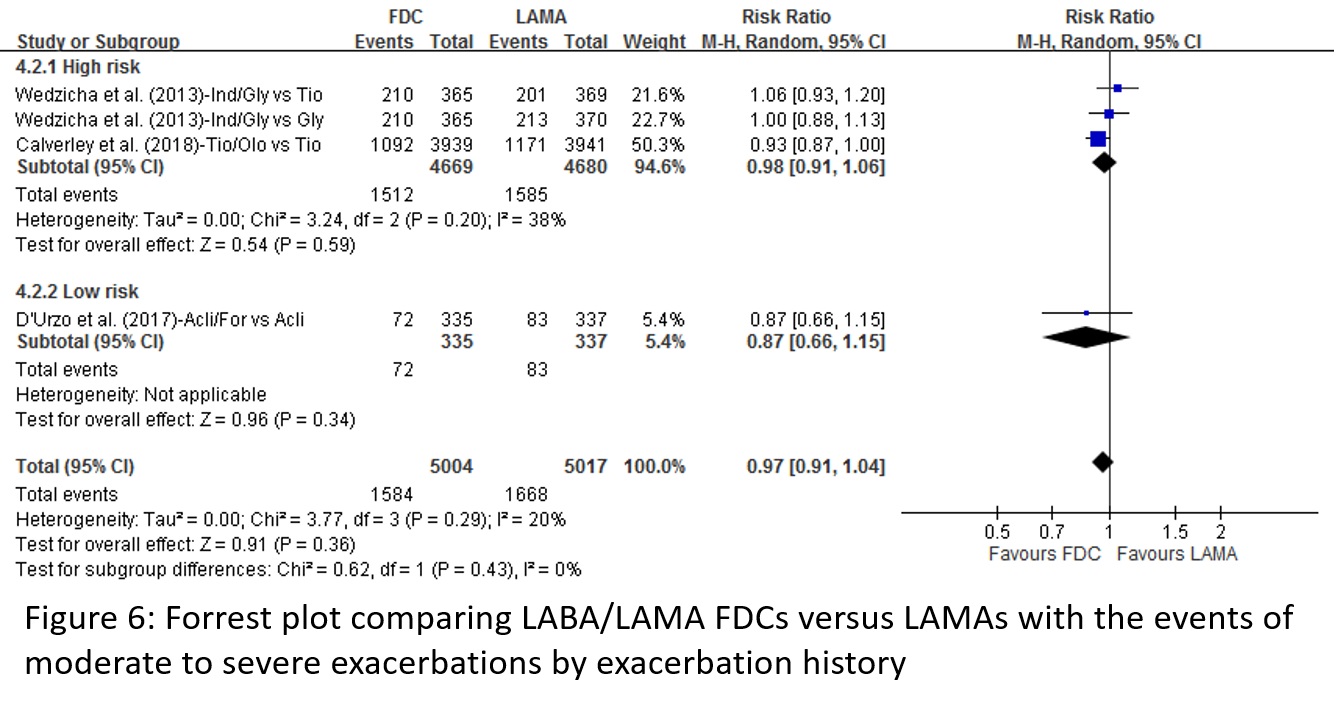

Supplement: Supplemental_Figures – Supplemental material for LABA/LAMA fixed-dose combinations versus LAMA monotherapy in the prevention of COPD exacerbations: a systematic review and meta-analysis [file Supplemental_Figures.zip › Figure S6.jpg]

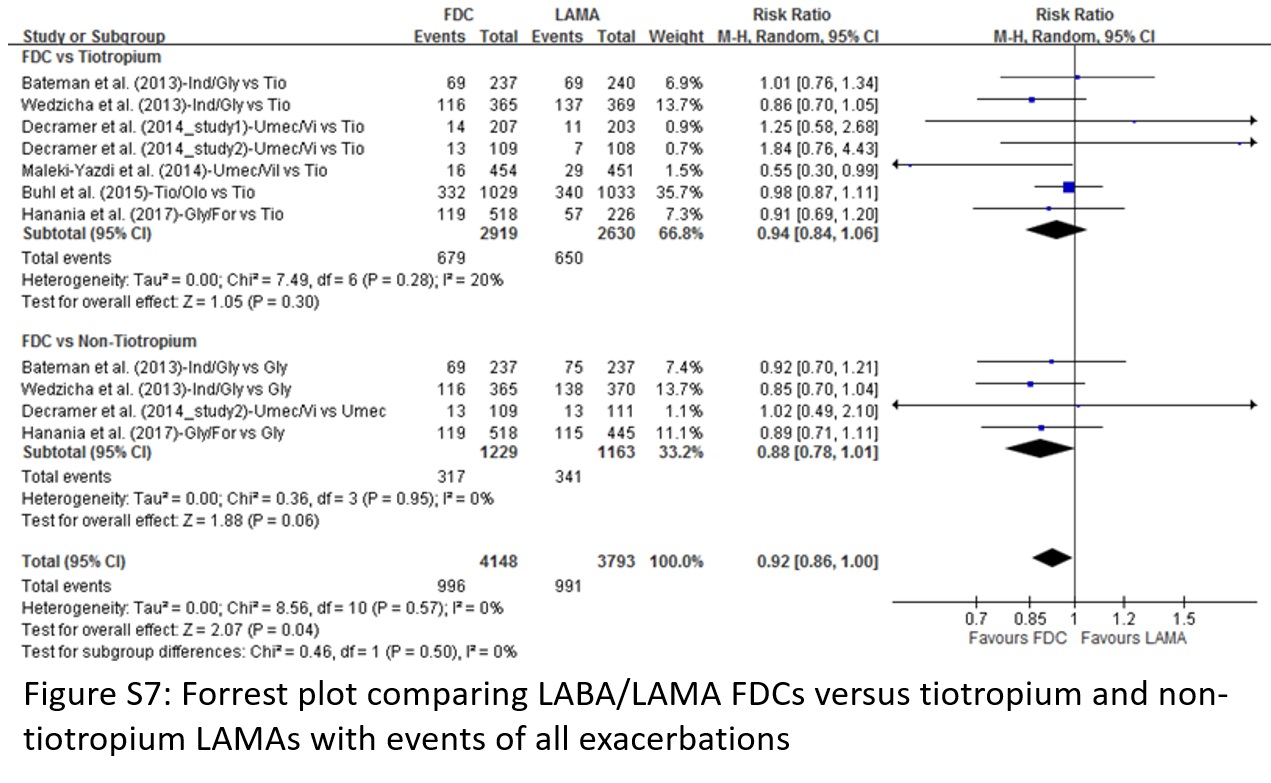

Supplement: Supplemental_Figures – Supplemental material for LABA/LAMA fixed-dose combinations versus LAMA monotherapy in the prevention of COPD exacerbations: a systematic review and meta-analysis [file Supplemental_Figures.zip › Figure S7.jpg]

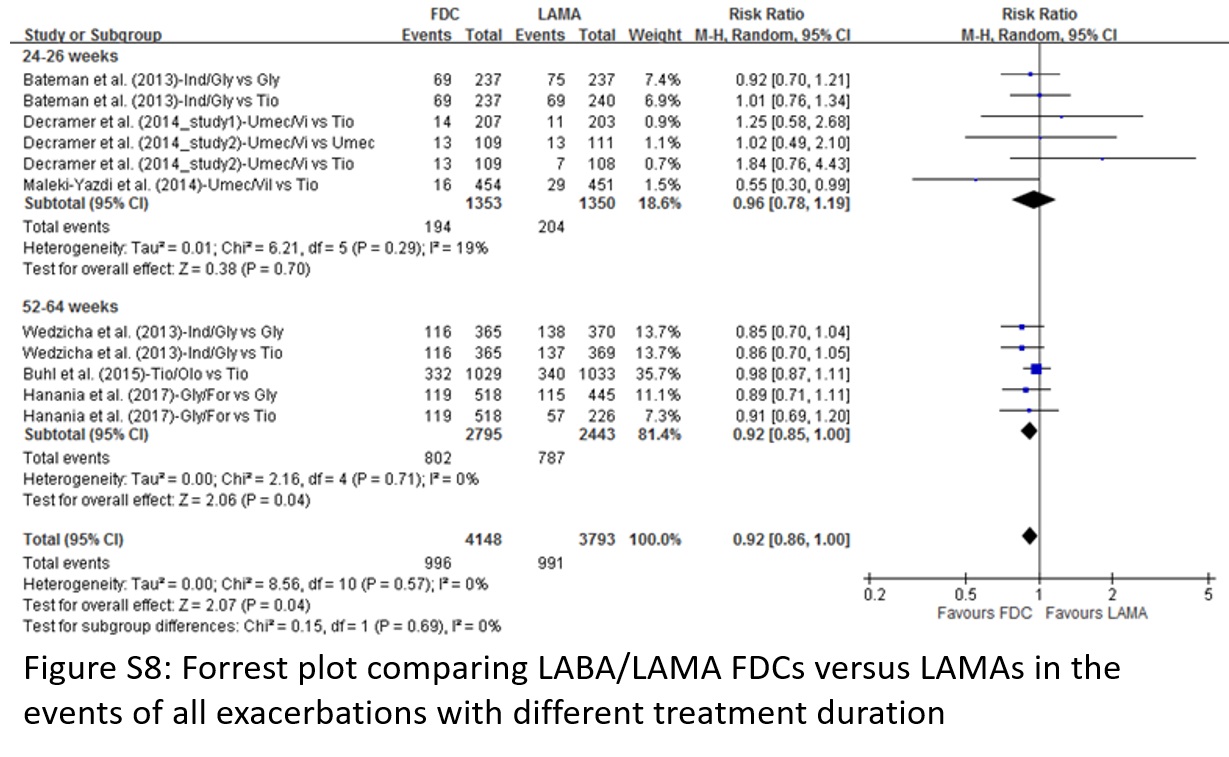

Supplement: Supplemental_Figures – Supplemental material for LABA/LAMA fixed-dose combinations versus LAMA monotherapy in the prevention of COPD exacerbations: a systematic review and meta-analysis [file Supplemental_Figures.zip › Figure S8.jpg]

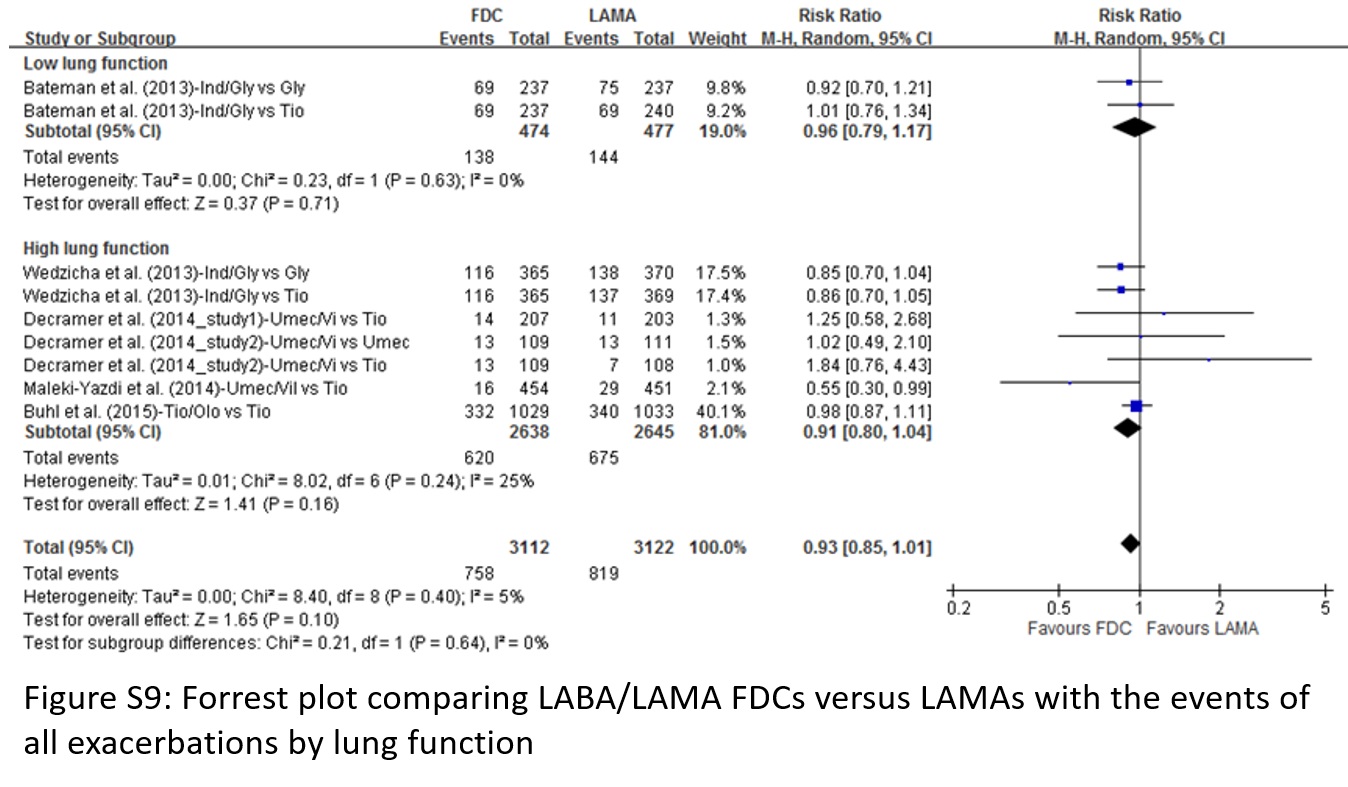

Supplement: Supplemental_Figures – Supplemental material for LABA/LAMA fixed-dose combinations versus LAMA monotherapy in the prevention of COPD exacerbations: a systematic review and meta-analysis [file Supplemental_Figures.zip › Figure S9.jpg]
