# Supplementary material for: LABA/LAMA fixed-dose combinations versus LAMA monotherapy in the prevention of COPD exacerbations: a systematic review and meta-analysis
Source: Ther Adv Respir Dis. 2020 Jul 9;14:1753466620937194. doi: 10.1177/1753466620937194 (PMC7350046; doi:10.1177/1753466620937194)
Supplement: Author_Response_1 – Supplemental material for LABA/LAMA fixed-dose combinations versus LAMA monotherapy in the prevention of COPD exacerbations: a systematic review and meta-analysis [file Author_Response_1.pdf]

Author Response 1:

Reviewer#1 comments

Methods:

Point 1

- Authors should describe their detailed search strategies. It is very surprising that they identified more manuscripts from the Cochrane Library, less from Pubmed and even less from Embase.

Answer:

Our detailed search strategies as below, fit final results in the PRISMA flow diagram.

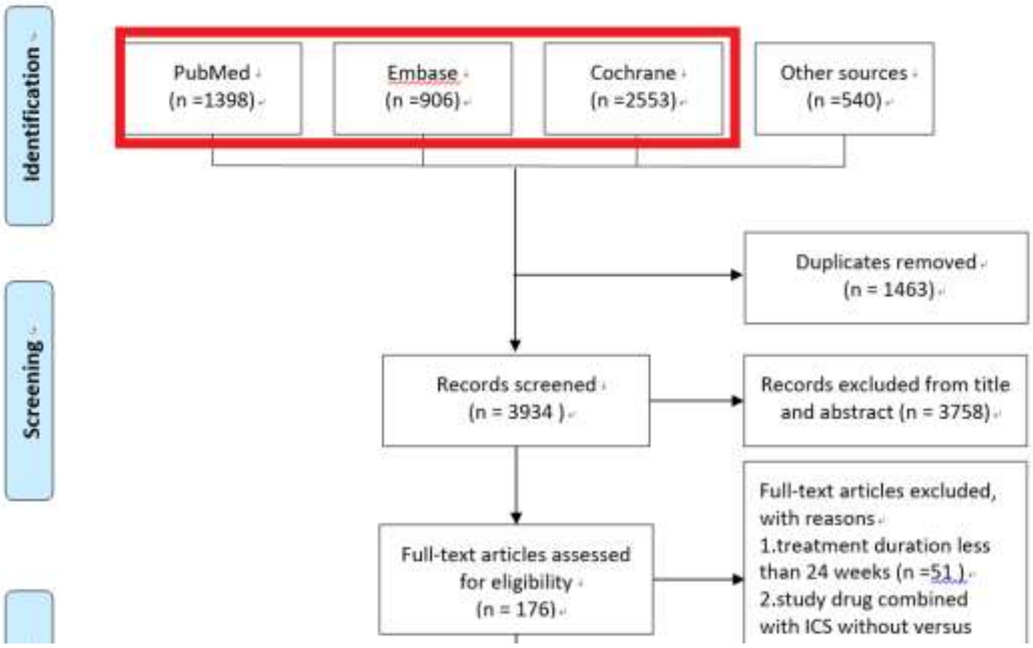

1. PubMed

NO.	key word	query	results
1#	"COPD" OR "Chronic obstructive pulmonary disease" OR "Pulmonary disease, chronic obstructive" OR "chronic obstructive lung disease" OR "chronic obstructive airway	"COPD" OR "Chronic obstructive pulmonary disease" OR "Pulmonary disease, chronic obstructive" OR "chronic obstructive lung disease" OR "chronic obstructive airway	85654

	disease" OR "chronic airway obstructive" OR "COAD" OR " Airflow obstruction, chronic" OR "chronic airflow obstructions" OR "obstructive lung disease" OR "obstructive pulmonary disease" OR "Lung disease, obstructive" OR "pulmonary disease, obstructive"	disease" OR "chronic airway obstructive" OR "COAD" OR " Airflow obstruction, chronic" OR "chronic airflow obstructions" OR "obstructive lung disease" OR "obstructive pulmonary disease" OR "Lung disease, obstructive" OR "pulmonary disease, obstructive" Filters: Full text, Randomized Controlled Trial	
2#	1# AND"single bronchodilator" OR "Dual bronchodilator" OR "LAMA" OR "LAMA combined LABA" OR "Glycopyrronium" OR "Tiotropium" OR "Umeclidinium" OR "aclidinium" OR "Olodaterol"	("COPD" OR "Chronic obstructive pulmonary disease" OR "Pulmonary disease, chronic obstructive" OR "chronic obstructive lung disease" OR "chronic obstructive airway disease" OR "chronic airway obstructive" OR "COAD" OR " Airflow obstruction, chronic" OR "chronic airflow obstructions" OR "obstructive lung disease" OR "obstructive pulmonary disease" OR "Lung disease, obstructive" OR "pulmonary disease, obstructive") OR ("single bronchodilator" OR "Dual bronchodilator" OR "LAMA" OR "LAMA combined LABA" OR "Glycopyrronium" OR "Tiotropium" OR "Umeclidinium" OR "aclidinium" OR "Olodaterol") Filters: Full text, Randomized Controlled Trial	**1398**

2. Embase

NO.	key word	query	results
1#	"COPD" OR "Chronic obstructive pulmonary disease" OR	('copd' OR 'chronic obstructive pulmonary disease' OR	6309

	“Pulmonary disease, chronic obstructive” OR “chronic obstructive lung disease” OR “chronic obstructive airway disease” OR “chronic airway obstructive” OR “COAD” OR “ Airflow obstruction, chronic” OR “chronic airflow obstructions” OR “obstructive lung disease” OR “obstructive pulmonary disease” OR ”Lung disease, obstructive” OR “pulmonary disease, obstructive” AND RCT	'pulmonary disease, chronic obstructive' OR 'chronic obstructive lung disease' OR 'chronic obstructive airway disease' OR 'chronic airway obstructive' OR 'coad' OR 'airflow obstruction, chronic' OR 'chronic airflow obstructions' OR 'obstructive lung disease' OR 'obstructive pulmonary disease' OR 'lung disease, obstructive' OR 'pulmonary disease, obstructive') AND [randomized controlled trial]/lim	
2#	1# AND“single bronchodilator” OR “Fixed-dose dual bronchodilator” OR “Dual bronchodilation” OR “Dual bronchodilator therapy” OR “long-acting muscarinic antagonist and Long-acting β2-agonist” OR “LAMA combined LABA” OR “long-acting muscarinic antagonist combined long-acting beta agonist inhalers” OR “long-acting muscarinic antagonist” OR “LAMA” OR “Glycopyrronium” OR “Tiotropium” OR “Umeclidinium” OR “aclidinium” OR “Olodaterol” OR “indacaterol/glycopyrronium” OR “umeclidinium/vilanterol” OR “Tiotropium/Olodaterol” OR “aclidinium/formoterol” AND RCT	('copd' OR 'chronic obstructive pulmonary disease' OR 'pulmonary disease, chronic obstructive' OR 'chronic obstructive lung disease' OR 'chronic obstructive airway disease' OR 'chronic airway obstructive' OR 'coad' OR 'airflow obstruction, chronic' OR 'chronic airflow obstructions' OR 'obstructive lung disease' OR 'obstructive pulmonary disease' OR 'lung disease, obstructive' OR 'pulmonary disease, obstructive') AND ('single bronchodilator' OR 'fixed-dose dual bronchodilator' OR 'dual bronchodilation' OR 'dual bronchodilator therapy' OR 'long-acting muscarinic antagonist and long-acting β2-agonist' OR 'lama combined laba' OR 'long-acting muscarinic antagonist combined long-acting beta agonist inhalers' OR	**906**

		'long-acting muscarinic antagonist' OR 'lama' OR 'glycopyrronium' OR 'tiotropium' OR 'umeclidinium' OR 'aclidinium' OR 'olodaterol' OR 'indacaterol/glycopyrronium' OR 'umeclidinium/vilanterol' OR 'tiotropium/olodaterol' OR 'aclidinium/formoterol') AND [randomized controlled trial]/lim	

3. Cochrane

NO.	key word	query	results
1#	“COPD” OR “Chronic obstructive pulmonary disease” OR “Pulmonary disease, chronic obstructive” OR “chronic obstructive lung disease” OR “chronic obstructive airway disease” OR “chronic airway obstructive” OR “COAD” OR “ Airflow obstruction, chronic” OR “chronic airflow obstructions” OR “obstructive lung disease” OR “obstructive pulmonary disease” OR ”Lung disease, obstructive” OR “pulmonary disease, obstructive” AND Trials	“COPD” OR “Chronic obstructive pulmonary disease” OR “Pulmonary disease, chronic obstructive” OR “chronic obstructive lung disease” OR “chronic obstructive airway disease” OR “chronic airway obstructive” OR “COAD” OR “ Airflow obstruction, chronic” OR “chronic airflow obstructions” OR “obstructive lung disease” OR “obstructive pulmonary disease” OR ”Lung disease, obstructive” OR “pulmonary disease, obstructive” in All Text - (Word variations have been searched)	19010
2#	1# AND“single bronchodilator” OR “Dual bronchodilator” OR “LAMA” OR “LAMA combined LABA” OR “Glycopyrronium” OR “Tiotropium” OR “Umeclidinium” OR “aclidinium” OR “Olodaterol” AND Trials	“COPD” OR “Chronic obstructive pulmonary disease” OR “Pulmonary disease, chronic obstructive” OR “chronic obstructive lung disease” OR “chronic obstructive airway disease” OR “chronic airway obstructive” OR “COAD” OR	**2553**

		" Airflow obstruction, chronic" OR "chronic airflow obstructions" OR "obstructive lung disease" OR "obstructive pulmonary disease" OR "Lung disease, obstructive" OR "pulmonary disease, obstructive" in All Text AND "single bronchodilator" OR "Dual bronchodilator" OR "LAMA" OR "LAMA combined LABA" OR "Glycopyrronium" OR "Tiotropium" OR "Umeclidinium" OR "aclidinium" OR "Olodaterol" in All Text - (Word variations have been searched)	

Point 2

- Was the SR protocol submitted to the PROSPERO register prospectively? It appears the study was started in April 2019 and systematic searches were conducted in August 2019 (3 months prior to registration).

Answer:

Indeed, we forgot to submit this systematic review for registration in PROSPERO initially, until the conduction of meta-analysis. The actual start of systematic searches was in April and our registration to PROSPERO was completed in early Aug 2019 (as below). However, the PROSPERO review is still ongoing

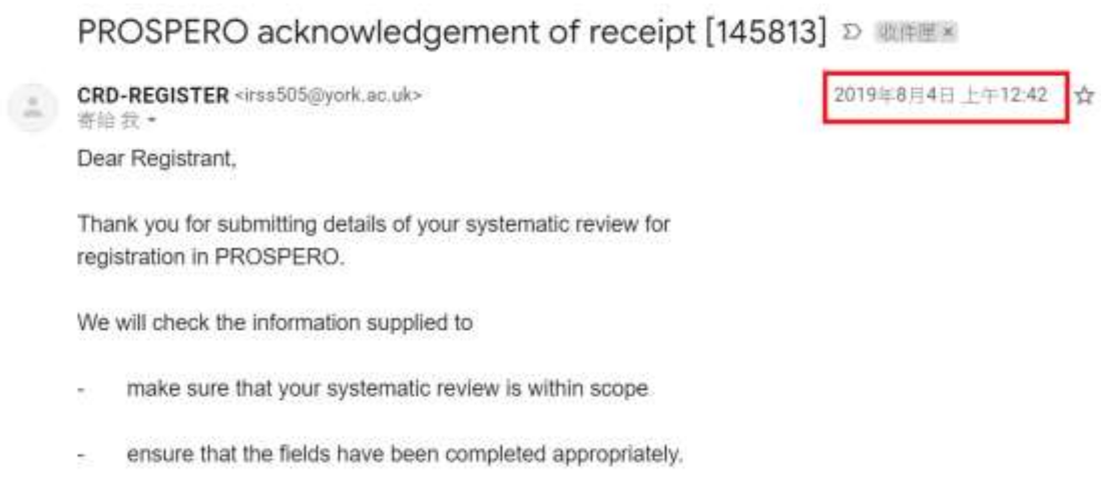
PROSPERO acknowledgement of receipt [145813] 收件匣 ×

CRD-REGISTER <irss505@york.ac.uk> 2019年8月4日 上午12:42
寄給我

Dear Registrant,

Thank you for submitting details of your systematic review for registration in PROSPERO.

We will check the information supplied to

- make sure that your systematic review is within scope
- ensure that the fields have been completed appropriately.

Point 3

- "For any three-arm trials (e.g., indacaterol/glycopyrronium versus glycopyrronium versus tiotropium), each pairwise comparison (i.e., indacaterol/glycopyrronium versus glycopyrronium, and indacaterol/ glycopyrronium versus tiotropium) was used in the meta-analysis by dividing the sample size in half". The sample of the control group should be divided in half, not the overall sample size. Please revise

Answer:

Yes, we did. We also divided the control group in our analysis. We amended our words to be more clear "by dividing the sample size in half to match the total sample size when adding together."

Point 4

- "All systematic review protocols were registered on PROSPERO with a publicly available database". Please rephrase.

Answer:

We have rephrased the sentence to "This systematic review protocols have been submitted to PROSPERO."

Results

Point 1

- In figure 1, authors state they identified 1,463 records after duplicates removed, but then state that 3,934 were screened. Could they explain?

Answer:

Actually, we identified 1463 duplicates, so we delete 1463 items. We have amended our flow diagram to avoid misunderstanding.

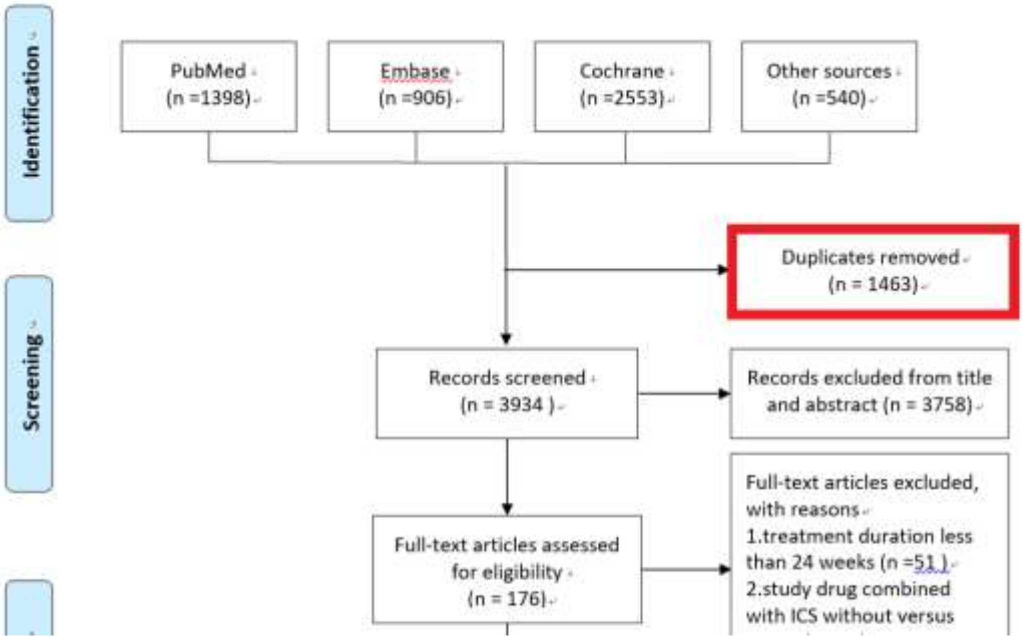

Point 2

- Subgroup analyses of studies evaluating high-/low- exacerbation risk populations are the most pertinent and are not presented in forest plots. Also, authors only conducted this subgroup analysis for the outcome "all exacerbations". They should evaluate it for all outcomes.

Answer:

The outcomes of interest in this study were the frequency of acute exacerbations including time to first exacerbation, rates of moderate to severe, severe, and all exacerbations. However, not all studies provide or measure these outcomes. We tried to analyse other exacerbation outcomes but only two studies evaluating high-/low- exacerbation risk populations (Wedzicha et al. 2013 [high risk] and Decramer et al. 2014[low risk]) provided "time to first exacerbation" as one measure outcome, and 3 studies (Wedzicha et al. 2013, Calverley et al. 2018 [high risk] and D'Urzo et al. 2017 [low risk]) provided "moderate to severe exacerbations" as an outcome. These two analysis results as below, which demonstrated no statistical difference in both outcomes. We already added these two figures (figure S3 and S6) in the supplementary files.

Figure S3: Time to first exacerbation by exacerbation risk (history)

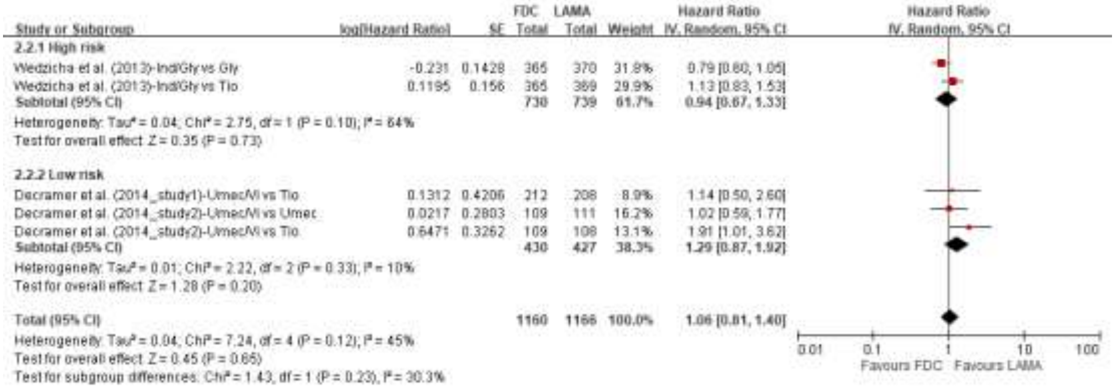

Figure S6: Risk of moderate to severe exacerbation by exacerbation risk (history)

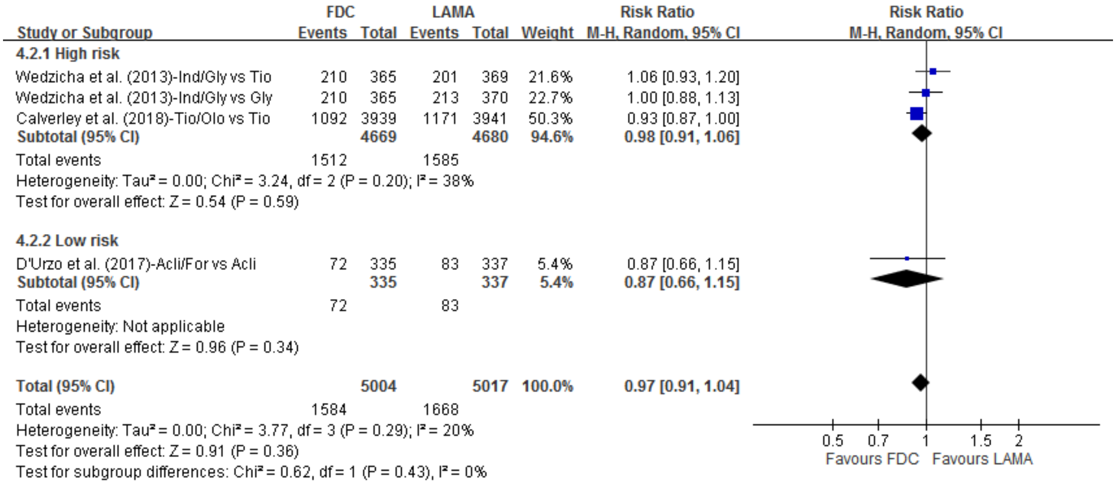

Point 3

- Actually, subgroup analyses are not clearly presented in the manuscript. Each for the subgroup analyses should be presented in the relevant section describing each outcome in the results.

Answer:

We have added available subgroup analyses outcomes in the results, including two figures regarding high-/low- exacerbation risk populations.

Discussion

Point 1

- Authors stated that only SPARK trial recruited patients with a history of exacerbations. However, that was also true for the DYNAGITO study. This will need to be taken in to consideration in the subgroup analyses.

Answer:

Our results demonstrated that only the incidence of "all exacerbation" (but not severe or moderate to severe exacerbation) events was lower in the LABA/LAMA group for COPD patients with a history of previous exacerbation. This result was truly from SPARK study only, because the DYNAGITO study did not provide outcomes of "all exacerbation". We have amended the sentence to be more clear and avoid misunderstanding.

Point 2

- The discussion needs to be revised. There is a bit of repetition and poor organization. Also, when judging the GOLD and NICE recommendations, authors should also consider the impact of LABA/LAMA vs LAMA monotherapy on symptoms and health status. Data on these may be found in the following meta-analyses: Miravitlles et al. Efficacy and safety of tiotropium and olodaterol in COPD: a systematic review and meta-analysis. Respir Res 2017. AND Ni et al. Combined aclidinium bromide and long-acting beta2-agonist for chronic obstructive pulmonary disease (COPD). Cochrane Database Syst Rev 2018.

Answer:

LABA/LAMA combinations have greater improvements in lung function and symptom scores compared to LABA or LAMA monotherapy, which is mentioned in the Introduction part. We have added these two references (Miravitlles et al. and Ni et al.) in the text (page 6, Introduction) accordingly.

Reviewer #2 comments:

Point 1

Abstract. The conclusion should be downgraded. It is now too strong compared to the poor results reported.

Answer:

We have amended the conclusion to "This study provides evidence that LABA/LAMA FDCs are marginally superior in the prevention of all exacerbations compared with LAMA monotherapy in patients with COPD."

Point 2

Results (page 11, line 43). It reads that two studies reported different results for the same population (Refs. 10 and 11) and only one was selected for inclusion. Can the authors expand on this? What was the difference? Which one was selected and why?

Answer:

These two articles reported different outcomes (safety outcomes in one and clinical outcomes in the other) from the same patients group, so we only selected one with clinical outcome (including exacerbations) for analysis in our study.

Point 3

Results. In general, more emphasis is required for analysis in patients at high risk for exacerbations. This sub-analysis should include patients with previous exacerbations (irrespective of lung function impairment).

Answer:

We have added our subgroup analyses outcomes in the results, including two figures (figure S3 and S6) regarding "time to first exacerbation" and "moderate to severe exacerbations" in high-/low- exacerbation risk (by exacerbation history) populations.

Point 4

Discussion (Page 15, line 19). GOLD is not a guideline, please rephrase.

Answer:

We have amended as "GOLD reports" in the text.

Point 5

Discussion (Page 18, line 16). The text reads that the only study including patients at high risk of exacerbations was SPARK, but DYNAGITO also included patients with at least one exacerbation the previous year and almost half of them with 2 or more or 1 severe.

Answer:

Our results demonstrated that only the incidence of “all exacerbation” (but not severe or moderate to severe exacerbation) events was lower in the LABA/LAMA group for COPD patients with a history of previous exacerbation. This result was truly from SPARK study only, because the DYNAGITO study did not provide outcomes of “all exacerbation”. We have amended the sentence to be more clear and avoid misunderstanding.

Point 6

Table 1. The description of DYNAGITO (Calverley et al) is wrong. It reads that 55.5% of patients had 0 exacerbations the previous year, but, by protocol, all included patents had to have at least 1 exacerbation the previous year. The 55.5% correspond to patients who had only one moderate exacerbation and the remaining 44.5% had either 2 or more moderate or at least 1 severe exacerbation. Therefore, when performing the sub-analysis of patients with high risk of exacerbations the authors must include the DYNAGITO participants and re-run the analysis.

Answer:

We have amended the description of the DYNAGITO study in table 1. We included DYNAGITO study as high-exacerbation risk populations and re-run subgroup analyses outcomes, but only “moderate to severe exacerbations” is available in the DYNAGITO study (figure S6).
